# Anti-fibrotic Actions of Equine Interleukin-10 on Transforming Growth Factor-Beta1-Stimulated Dermal Fibroblasts Isolated From Limbs of Horses

**DOI:** 10.3389/fvets.2020.577835

**Published:** 2020-09-18

**Authors:** Lyn M. Wise, Gabriella S. Stuart, Kevalee Sriutaisuk, Brooke R. Adams, Christopher B. Riley, Christine L. Theoret

**Affiliations:** ^1^Department of Pharmacology and Toxicology, University of Otago, Dunedin, New Zealand; ^2^School of Veterinary Science, Massey University, Palmerston North, New Zealand; ^3^Département de Biomedecine Vétérinaire, Faculté de Médecine Vétérinaire, Université de Montréal, Montreal, QC, Canada

**Keywords:** interleukin-10, transforming growth factor-beta1, fibroblast, horse, exuberant granulation tissue

## Abstract

Fibroproliferative disorders occur in both humans and horses following skin injury. In horses, wound healing on the limb is often complicated by the formation of fibroproliferative exuberant granulation tissue, characterized by persistent expression of pro-fibrotic transforming growth factor-beta1 (TGF-β1) and deficient expression of anti-inflammatory interleukin-10 (IL-10). IL-10 has been shown to directly modulate fibrotic gene expression in human fibroblasts, so we hypothesized that equine IL-10 (eIL-10) may exert similar anti-fibrotic effects on equine dermal fibroblasts. Cell-lines were created from the limb skin of six individual horses. Recombinant eIL-10 was produced and purified, and its effects on the cells investigated in the presence and absence of equine TGF-β1 (eTGF-β1). Myofibroblast differentiation and collagen production were examined using immunofluorescent cytometry, cell contractility in a collagen gel assay, and fibrotic gene expression using quantitative PCR. In response to eTGF-β1, fibroblasts increased in contractility and expression of alpha-smooth muscle actin, collagen types 1 and 3, and matrix metalloproteinase 1, 2, and 9. Equine IL-10 limited cell contractility and production of alpha-smooth muscle actin and type 3 collagen, and decreased mRNA levels of *eCol3a1* and *eMMP9*, while increasing that of *eMMP1*. Opposing effects on *eTGF-*β*R3* and *eIL-10R1* gene expression were also observed, with mRNA levels decreasing following eTGF-β1 treatment, and increasing with eIL-10 treatment. These findings indicate that eIL-10 limits the pro-fibrotic effects of eTGF-β1, potentially through the modulation of fibrotic and receptor gene expression. Further investigations are warranted to assess the therapeutic utility of eIL-10 in the treatment of exuberant granulation tissue.

## Introduction

The repair of damaged tissues is crucial to recovery and survival (1). This fundamental biological process ensures the replacement of cells that have been damaged or lost due to trauma, and the restoration of the function of the skin. In response to injury, chemotactic stimuli trigger the recruitment of immune cells, including neutrophils and macrophages. These infiltrating immune cells produce pro-inflammatory cytokines and growth factors that trigger the activation of myofibroblasts, the main effectors of tissue contraction and remodeling during repair. Under ideal physiological conditions, remodeling leads to regeneration of the lost tissue and minimal scarring. The healing process can become pathogenic if it is left unchecked or is otherwise aberrant. In this case, chronic inflammation disrupts the balance between synthesis and degradation of extracellular matrix (ECM) components, and the continuously activated myofibroblasts produce excessive ECM, leading to the replacement of parenchymal tissue with connective tissue (2). Chronic pathogenic remodeling leads, ultimately, to the destruction of normal organ architecture and the consequent decline of its function, and is known as fibrosis. Fibrotic changes can occur in all tissues and organ systems, and result in fibroproliferative disorders (3).

Skin wounds in horses occur frequently and are of significant financial and welfare concern to the equine industry. In this species, wounds generally must heal by secondary intention as excessive tissue loss and environmental exposure to contaminants precludes primary closure. In the USA and the UK, wounds are the most common medical condition in horses (4, 5). In Australia and New Zealand, veterinarians ranked wounds as the second most frequent cause of death or euthanasia (6, 7). In addition, wounds that occur on the distal extremity of the limb, as opposed to those occuring on the body, are often complicated by a fibroproliferative disorder known clinically as “proud flesh.” This presents as exuberant granulation tissue (EGT), an excessive deposition of granulation tissue within the wound bed that protrudes above the level of the surrounding skin and prevents wound contraction and re-epithelialisation (8).

EGT, present in limb wounds, is characterized by a protracted inflammatory response, with deficient expression of interleukin (IL)-10, and persistent expression of pro-fibrotic transforming growth factor-beta1 (TGF-β1) and its receptors TGF-βR1 and TGF-βR2 and type 1 collagen, relative to body wounds (9–13). Compared with other species, horse limb wounds also suffer from impaired contraction which has been linked to the mitotic phenotype and chaotic organization of myofibroblasts observed within granulation tissue (9, 14). Many approaches have been used to treat EGT, but the most successful approaches combine excision of the protruding granulation tissue with therapies that treat chronic inflammation (15). New methods aimed at preventing EGT formation are, however, being sought to reduce the need for surgical intervention and to curb the high rates of recurrence.

Soluble mediators such as growth factors and cytokines have a critical influence on repair processes. IL-10 is one such mediator that exerts pleiotropic effects during tissue repair. IL-10 plays a major role in the limitation and termination of the inflammatory response. Application of the IL-10 gene or protein to adult mouse skin decreases the expression of pro-inflammatory mediators, reducing infiltration of inflammatory cells into wound tissue (16–18). IL-10 also inhibits fibrosis, contributing to scar-free healing in the mouse embryo, and restoring the normal ECM architecture in scars of adult mice (19). *In vitro* studies have also demonstrated IL-10's anti-fibrotic effects on human dermal fibroblasts, including suppression of TGF-β1-induced production of alpha-smooth muscle actin (αSMA) contractile stress fibers and myofibroblast differentiation, inhibition of type 1 and type 3 collagen (*Col1*α*2* and *Col3*α*1*) expression, and enhancement of matrix metalloproteinase (MMP) 1 expression and cell motility (20–22). Consistent with these findings, IL-10 administration to human incisional wounds improved the macroscopic appearance of scars (23). Short-term administration of a viral IL-10 protein (in combination with viral vascular endothelial growth factor-E) also dampened immune cell infiltration, and accelerated EGT resolution in experimentally-induced limb wounds in horses (24).

Given that IL-10 from different species acts in a similar manner to modulate wound healing responses, we hypothesized that equine IL-10 (eIL-10) would also exert anti-fibrotic effects. To investigate this hypothesis we created fibroblast cell-lines from the limb skin of six individual horses. We then produced and purified recombinant equine IL-10 and examined its effect on the dermal fibroblast cells, specifically on their contractility, differentiation capacity, production of collagen subtypes, and fibrotic gene expression, in the presence and absence of equine TGF-β1 (eTGF-β1). The concentrations of eIL-10 and eTGF-β1 used were equivalent to those reported to be effective for the human proteins (20, 21). The overall objective was to ascertain if eIL-10 could inhibit the pro-fibrotic effects of eTGF-β1, and as such could offer therapeutic benefit in the prevention of EGT in horses.

## Materials and Methods

### Equine Skin Samples

Skin samples were obtained from six healthy horses that were euthanised at their owner's request for reasons unrelated to this protocol. Multiple biopsies (8–12 samples; 4 cm^2^) were obtained per horse, and placed in Dulbecco's modified Eagle's medium (DMEM) with 5% fetal bovine serum (FBS), 50 μg/mL gentamycin and 2.5 μg/mL amphotericin B, then stored for 24 h at 4°C.

### Equine Cell Culture

Cells were extracted from the skin samples using an explant method (12). Skin samples were cleaned with 10% povidone-iodine and 70% ethanol then fat and blood vessels removed. Biopsies were cut into 1 mm^2^ fragments then placed upright 1 cm apart in culture wells previously coated with 0.1% gelatin for 30 min. For 7 days, the explants were partially submerged in DMEM containing 20% FBS, 5 μg/mL ascorbic acid, 100 I.U/ml penicllin, 100 μg/ml streptomycin and kanamycin, 50 μg/mL gentamycin, and 2.5 μg/mL amphotericin B, then incubated at 37°C/5% CO_2_. At that point, cell colonies were lifted, and combined to generate a single cell line for each individual horse. Cell lines were then maintained in growth media, DMEM containing 10% FBS and antibiotics, at a density of 1 × 10^4^ – 1 × 10^5^ cells/mL, with subculturing at 70–80% confluency. Cells were used in assays at passage 4–5.

### Production of Recombinant Equine IL-10

A DNA fragment containing nucleotides 1-534 of eIL-10 (NCBI Reference Sequence: NM_001082490.1) was prepared by polymerase chain reaction (PCR) using the Expand High Fidelity PCR System (Roche, Basel, Switzerland) and a cDNA template. The cDNA was generated from equine dermal fibroblasts using the TriRNA Pure Kit (Geneaid, New Taipei City, Taiwan) followed by DNase treatment (Quanta Bio, Beverly, MA) and the SuperScript III First-Strand Synthesis System (Invitrogen, Carlsbad, CA, USA). The primers used to amplify eIL-10 and to incorporate a C-terminal FLAG octapeptide were 5′AAAGGATCCCACCATGCACAGCTCAGCACTGCTATGTTAC (pA:eIL-10F) and 5′AAAGGCGCGCCGTTTTTCATCTTCGTTGTCATATAGGCTTC (pA:eIL-10R). The fragment was inserted into the BamHI and AscI sites generated in the expression vector pAPEX-3-FLAG. FLAG-tagged eIL-10 was expressed by transfection of 293 EBV-encoded nuclear antigen cells with the vector using FuGENE-6 (Promega, Madison, WI, USA), then purified by affinity chromatography with anti-FLAG M2 affinity gel (Sigma-Aldrich, St Louis, MO, USA). The purified protein was then resolved by SDS-PAGE, stained with Coomassie blue, and bands quantitated using a densitometer and the Quantity One program (Bio-Rad Laboratories). Recombinant FLAG-tagged human (h) IL-10 was produced in a similar manner, as previously described (18).

### Protein Deglycosylation

To compare the glycosylation states of eIL-10 and hIL-10, purified protein (500 ng) was diluted in 0.05 M sodium phosphate (pH7) containing 0.1% sodium dodecyl sulfate and 20 mM 2-mercaptoethanol and boiled for 5 min. The mixture was cooled on ice and Tween 20 was added to 0.75%. Either five units of N-glycosidase F (Roche, Mannheim, Germany) or no enzyme was added, then the mixtures incubated at 37°C for 3 h. The deglycosylated proteins were then resolved by SDS-PAGE and visualized by western blotting using an anti-FLAG Peroxidase antibody (M2, mouse monoclonal, Sigma-Aldrich).

### Competitive Displacement IL-10 Receptor Binding Assay

A modified enzyme-linked immunosorbant assay (ELISA) was used to assess binding of the recombinant IL-10s for IL-10R1. Maxisorp 96-well immunoplates (Nunc, Roskilde, Denmark) were incubated with 400 ng/mL of hIL-10 in coating buffer (15 mm Na^2^CO_3_, 35 mm NaHCO_3_, pH 9.6) at 4°C for 16 h and blocked with 1% bovine serum albumin (BSA) and 0.02% Tween 20 at 37°C for 45 min. Plates were washed between steps with wash buffer. Samples of hIL-10 or eIL-10, serially diluted in binding buffer (NaCl / Pi with 0.4% BSA and 0.02% Tween 20), were incubated with 300 ng/mL human IL-10R1–Fc chimeric protein (R&D Systems, Minneapolis, MN, USA) in non-absorbent plates at 25°C for 1 h. The mixture was then transferred to plates coated with hIL-10 and incubated at 25°C for 1 h to capture the unbound hIL-10R1–Fc protein. The captured IL-10R1–Fc protein was detected by biotinylated anti-human Ig (Dako, Glostrup, Denmark) and streptavidin-peroxidase (Sigma) and tetramethylbenzidine substrate reagent and quantified by measuring the absorbance at 450 nm. The percentage change in IL-10R1-Fc protein bound was then calculated relative to that detected in the absence of soluble IL-10.

### Immunofluorescent Analyses

Cells were seeded onto sterile coverslips in growth media at a density of 1 × 10^4^ cells/mL. Where indicated, the growth media was supplemented with 0–10 ng/mL eTGF-β1 and/or 0–100 ng/mL eIL-10. After 3 days incubation at 37°C/5% CO_2_, cells were fixed in ice cold 100% methanol, then permeabilised with 10% Tween20, for 45 min each. To analyse phenotype, cells were incubated with antibodies against vimentin (D21H3, XP® AlexaFluor®488 conjugate, Cell Signaling Technology, Danvers, MA, USA; 1:50 dilution), αSMA (1A4, AlexaFluor®594 conjugate, Abcam, Cambridge, UK; 1:50 dilution), chondroitin sulfate proteoglycan 4 (NG2, rabbit polyclonal, Abcam #ab129051; 1:200 dilution), platelet endothelial cell adhesion molecule (CD31, M-20, goat polyclonal, sc-1506, Santa Cruz Biotechnologies, Dallas, TX, USA; 1:200 dilution), preadipocyte factor-1 (Pref-1/Dlk-1, 3A10, mouse monoclonal, Abcam #ab119930; 1:200 dilution), claudin1 (CLDN1, EPRR18871, rabbit monoclonal, Abcam #ab211737; 1:200 dilution) in Tris-buffered saline containing 0.1% BSA for 2 h at room temperature (RT). To analyse collagen subtypes, cells were incubated with antibodies against Col 3 (rabbit polyclonal, Abcam #ab7778; 1:200 dilution) or Col 1 (rabbit polyclonal, Abcam #ab34710; 1:200 dilution) overnight at 4°C. Where necessary, a further 1 h incubation at RT was performed with AlexaFluor®594 or AlexaFluor®488-conjugated anti-rabbit, anti-mouse, and/or anti-goat antibodies (Invitrogen; 1:500 dilution). For the final 30 min, 4',6-diamidino-2-phenylindole (DAPI, 75 nM, Invitrogen) was added. Coverslips were mounted onto slides using SlowFade Gold (Invitrogen) and stored at 4°C. Stained cells were photographed at 20X magnification on an Eclipse Ni-E upright fluorescence microscope and NIS-Elements D Software (Nikon, Tokyo, Japan). Ten images were taken at random locations within each slide, and the number of stained cells within each image was counted using the Particle Analysis tool in FIJI (25) (available from https://imagej.net/Fiji).

### Collagen Gel Contraction Assay

Gels were prepared from 2 mg/ml collagen (col) 1 (rat tail; Sigma) in DMEM containing 10% FBS and antibiotics, with the addition of 0.1 M NaOH to pH 7.4. Cells were added at a density of 1 × 10^5^ cells/mL of gel. Cell-laden gel was then aliquoted (0.5 mL/well) into a 24-well plate and allowed to set for 2 h at at 37°C/5% CO_2_. Once set, the gels were carefully dislodged and floated in DMEM containing 10% FBS and antibiotics, and where indicated, 0–10 ng/mL eTGF-β1 and/or 0–100 ng/mL eIL-10. Prior to their release and during the 72 hr incubation, images of the gels were taken with a digital camera. The percentage change in gel area over time was then calculated from area measurements performed using FIJI using the external perimeter of each well as a standardized scale.

### Quantitative PCR Analyses

Cells were seeded at a density of 1 × 10^5^ cells/mL. Where indicated, the cell media was supplemented at the time of seeding with 10 ng/mL eTGF-β1 (R&D Systems, Minneapolis, MN, USA) and/or 10 ng/mL eIL-10. Cells were then incubated at 37°C/5% CO_2_. At indicated time points, cells (5 mL volumes) were harvested then RNA isolated and cDNA synthesized as described above. Quantitative PCR was conducted using cDNA equivalent of 5 ng RNA, 200 mM of each primer, and PerfeCTa SYBR Green SuperMix (Quanta Bio) on the Mic qPCR Cycler (Bio Molecular Systems, Sydney, Australia). Primer pairs and their relative efficiencies are provided in Supplementary Table 1. Gene expression was normalized to equine glyceraldehyde-3-phosphate dehydrogenase (*eGAPDH*) and to mock-treated cells.

### Statistical Analyses

Values are expressed as the mean ± standard error of the mean (SEM). One-way analysis of variance (ANOVA), with repeated measures corresponding to each cell line, was conducted for the gene expression, immunofluorescent and contraction analyses. Significant points of difference between means were also determined using Tukey's test following correction for multiple comparisons. Values of *p* < 0.05 were considered statistically significant.

## Results

### Creation of Dermal Fibroblast Cell Lines From the Limbs of Six Horses

**Figure 1:**
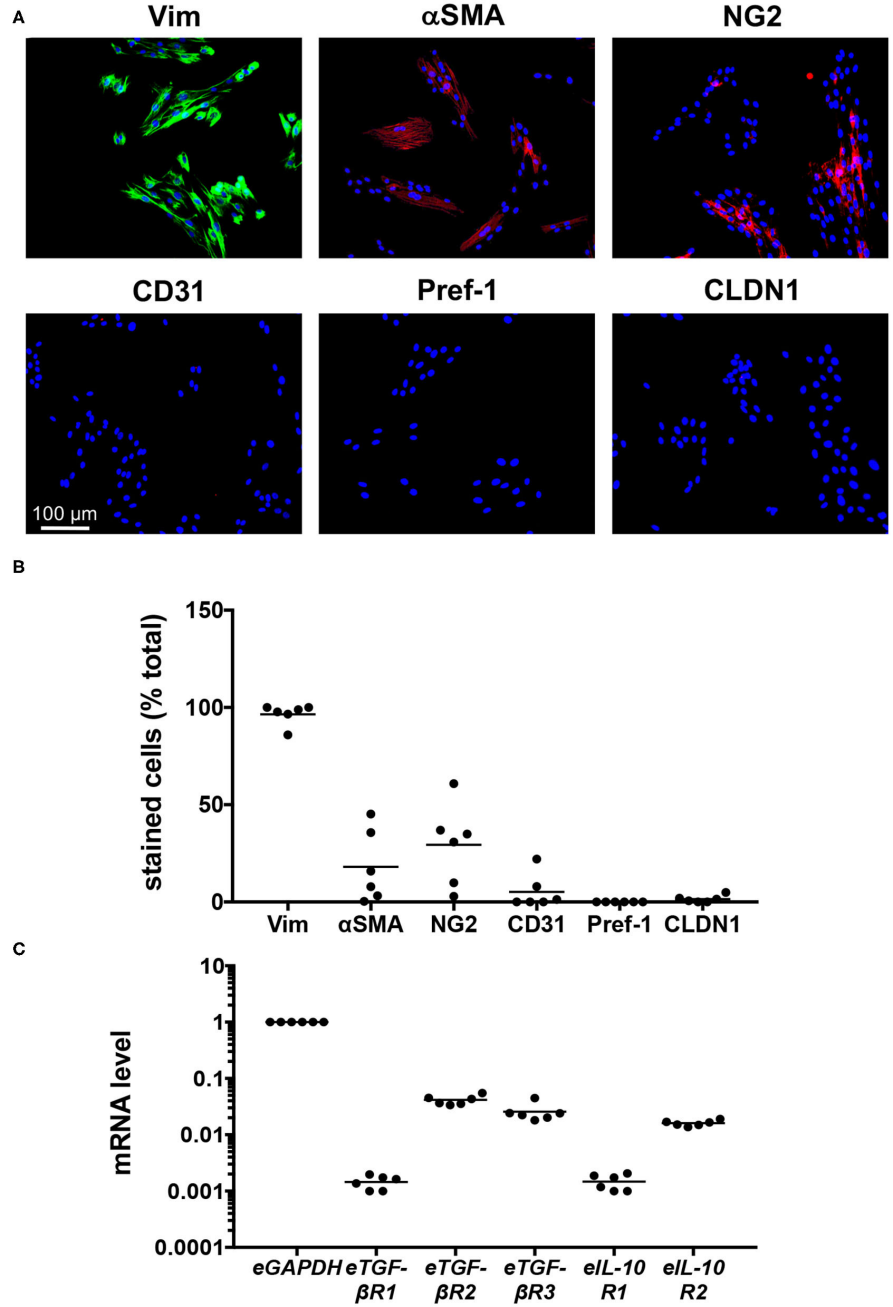
Characterization of six cell-lines created from limb skin of horses. **(A)** Images of cell-lines derived from an individual horse (H6), cultured for 72 h, then stained with DAPI and antibodies against vimentin (Vim), αSMA, NG2, CD31, Pref-1, and CLDN1. Scale is as indicated. **(B)** The percentage of DAPI-stained cells co-stained for the phenotypic markers. **(C)** Quantitative PCR was used to measure mRNA levels in cell-lines derived from individual horses, cultured for 24 h. The level of mRNA is relative to that of *eGAPDH*. Each circle represents a cell-line derived from an individual horse, while the line indicates the mean of the six cell-lines.

To establish their phenotype, cell lines created from the limb skin of the six individual horses were analyzed using immunofluorescence (Figure 1A), with the specificity of antibody staining established using equine skin (24) as well as isotype and secondary only controls (Supplementary Figure 1). The cell-lines were primarily of fibroblast origin, with 86–100% of cells staining positive for vimentin (Figure 1B). There was, however, variable expression of αSMA (1–45%), and NG2 (3–61%) across cell-lines, indicating the presence of myofibroblasts and pericytes, respectively (Figure 1B). Two cell lines showed the presence of endothelial cell-expressed CD31, while negligible expression of the keratinocyte and pre-adipocyte markers CLDN1 and Pref-1 was observed (Figure 1B). These findings indicate that the six equine dermal cell-lines are primarily fibroblast-like, and so can be refered to as equine dermal fibroblasts (EDF).

To establish whether the EDF cell-lines were likely to respond to treatment, quantitative PCR was used to evaluate their expression of the receptors for eTGF-β1 and eIL-10. For the most part, expression levels for each gene were consistent across cell lines, with mRNA levels for *eTGF-*β*R2, eTGF-*β*R3*, and *eIL-10R2* 20-60-fold lower than that of *eGAPDH*, and those for *eTGF-*β*R1* and *eIL-10R1* at least 500-fold lower (Figure 1C). As such the cells should be responsive to eTGF-β1 and eIL-10.

### Characterization of Recombinant Equine IL-10

**Figure 2:**
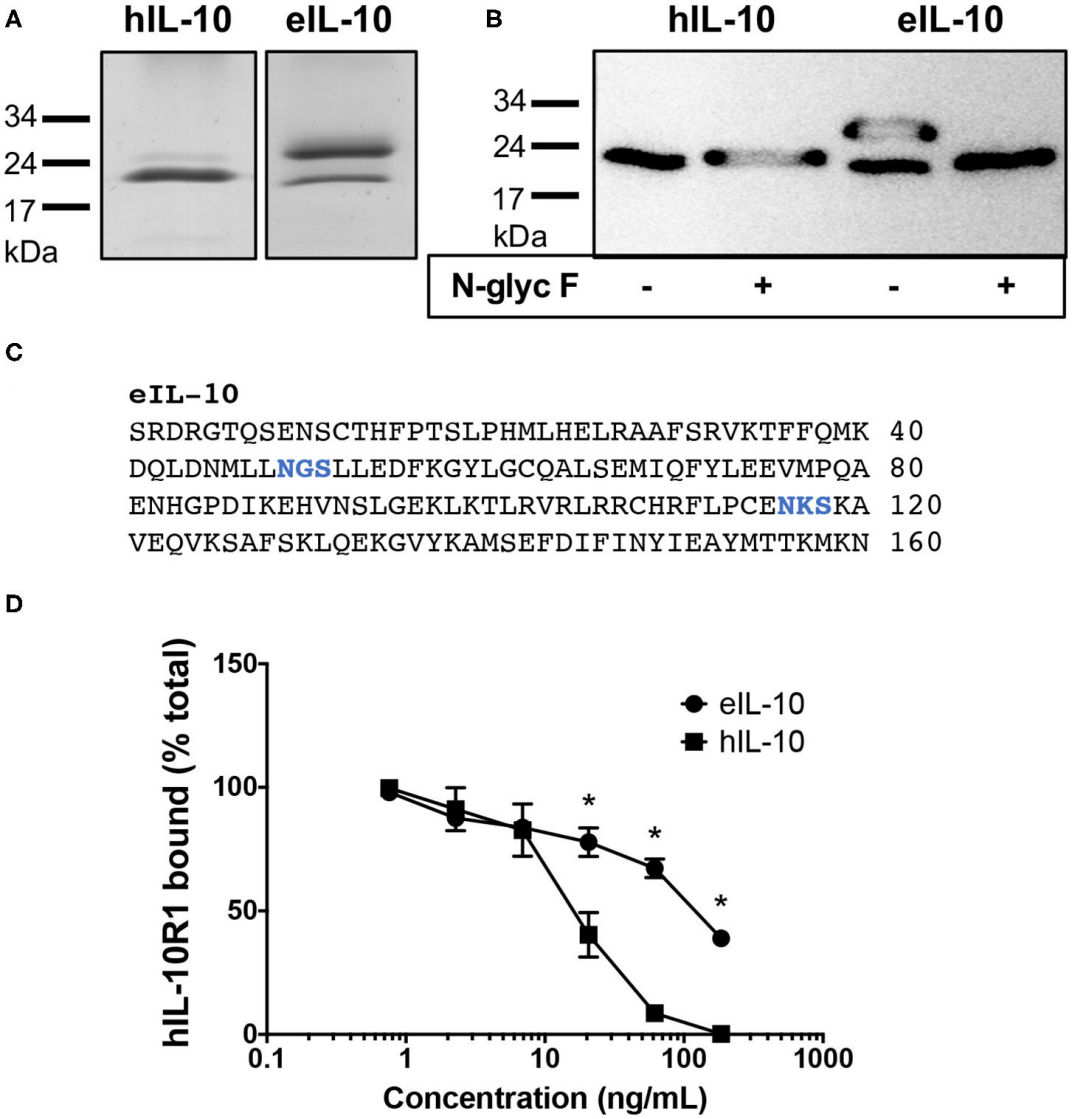
Characterization of recombinant equine IL-10. **(A)** Coomassie blue-stained SDS-Page gel showing bands for hIL-10 and eIL-10. **(B)** Anti-FLAG western blot showing bands for hIL-10 and eIL-10, with or without prior digestion by N-glycosidase F (N-glyc F). The positions of molecular mass markers are shown on the left. The 21 kDa proteins represent IL-10 monomers, while the 26 kDa form show a glycosylated form of eIL-10. **(C)** Protein sequence of mature eIL-10 protein showing putative N-linked glycosylation sites in blue, as determined using the NetNGlyc 1.0 Server at http://www.cbs.dtu.dk/services/NetNGlyc/ **(D)** Soluble hIL-10R1–Fc fusion protein was incubated with increasing concentrations hIL-10 or eIL-10. The mixture was then added to hIL-10-coated wells to capture free hIL-10R1-Fc, which was detected with a biotinylated anti-human Ig, streptavidin–HRP conjugate. The results are presented as the percentage of the maximal absorbance of hIL-10R1–Fc bound. Values are expressed as mean ± SEM (*n* = 3). Concentrations of eIL-10 with significantly different means from that of hIL-10 are indicated by an asterisk (*P* ≤ 0.05).

Recombinant eIL-10, tagged at the C terminus with the FLAG octapeptide, was expressed and purified. The protein was then identified using SDS-PAGE using Coomassie blue staining. Protein bands were observed at 21 kDa, consistent with its predicted size, and that reported for hIL-10 (Figure 2A). The additional protein band observed at 26 kDa suggests that eIL-10 can exist in a glycosylated form. Indeed, enzymatic digestion with an N-glycosidase F eliminated the 26 kDa band of eIL-10 (Figure 2B). Bioinformatic analyses indicate two putative N-linked glycosylation sites in the eIL-10 protein (Figure 2C). The hIL-10 is predicted to have one N-linked glycosylation sites, but digestion with N-glycosidase F had no impact on its size (Figure 2B). eIL-10 therefore differs in glycosylation from hIL-10 produced using the same expression system.

To establish whether the recombinant eIL-10 was capable of binding its receptor, the purified protein was tested for its ability to inhibit hIL-10 binding to an Fc chimeric protein containing the extracellular domain of hIL-10R1 using a competitive displacement ELISA. The human receptor was trialed in this assay, as it was not possible to source or to produce the purified equine receptor. eIL-10 shares 85% identity with the hIL-10, while eIL-10R1 shares 72% identity with the hIL-10R1. Preincubation of hIL-10 with hIL-10R1 inhibited the receptor binding to immobilized hIL-10 from 7 ng/mL (*P* ≤ 0.05, Figure 2D). eIL-10 was less potent than soluble hIL-10, but inhibited receptor binding from 20 ng/mL (*P* ≤ 0.05, Figure 2D). eIL-10 is therefore capable of binding the human receptor, and thus likely the equine equivalent.

### Equine IL-10 Inhibits eTGF-β1-Induced Contractility in EDFs

**Figure 3:**
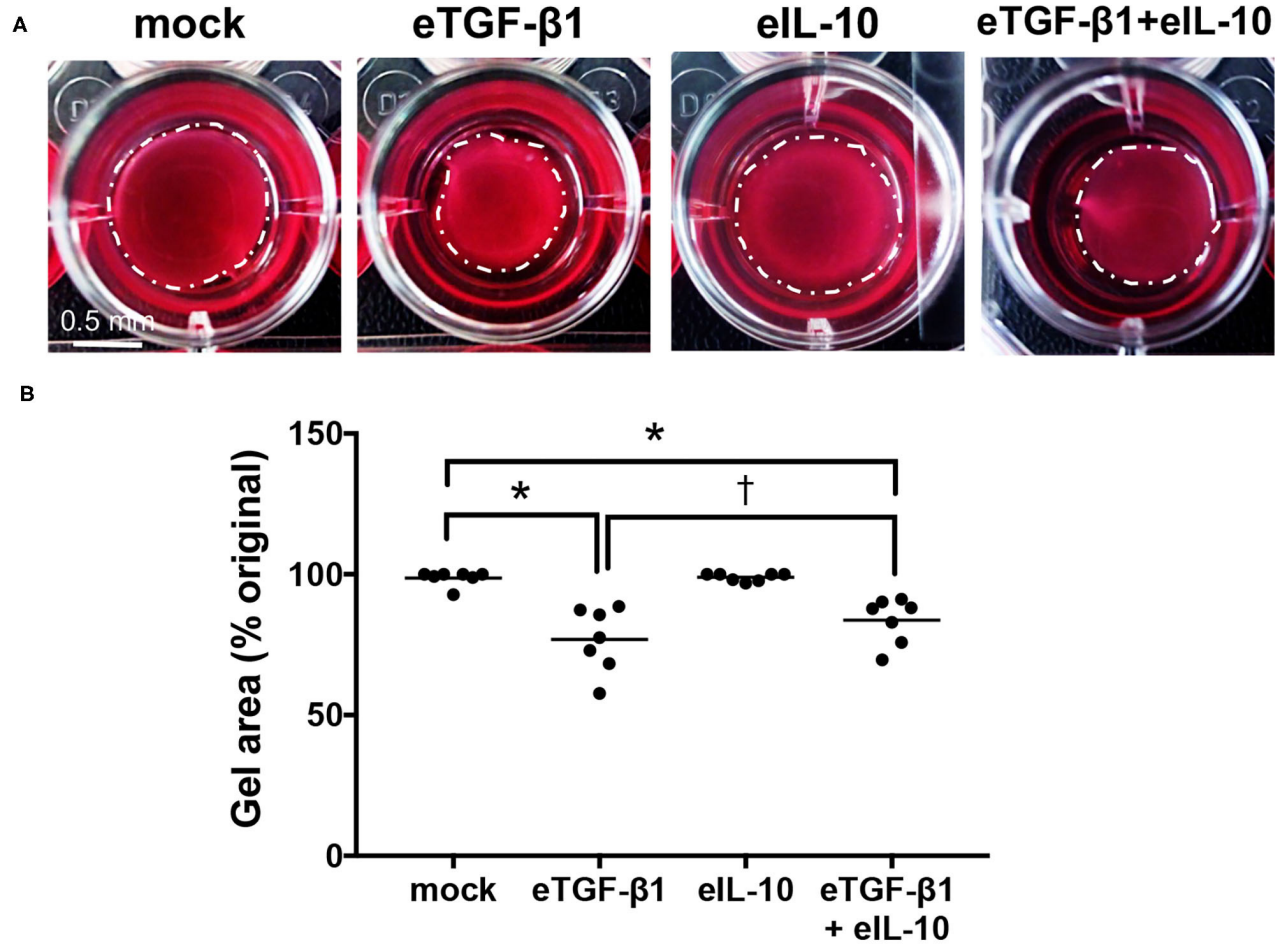
Equine IL-10 limits eTGF-β1-induced collagen contraction by EDFs. **(A)** Images of representative Col 1 gels containing cell-line H6, released then cultured for 72 h with eTGF-β1 (10 ng/mL) and/or eIL-10 (10 ng/mL). Scale is as indicated. **(B)** Gel area is presented as a percentage of the original size at the time of release. Each circle represents a cell-line derived from an individual horse, while the line indicates the mean of the six cell-lines. Means significantly different from that of mock or eTGF-β1-treated cells are indicated by an asterisk or cross, respectively (*P* ≤ 0.05).

The effects of eTGF-β1 and/or eIL-10 on ECM contraction by the EDF cell-lines were assessed using a collagen gel contraction assay (Supplementary Figure 2, Figure 3A). The assay was optimized using a single cell-line, with 10 ng/mL eTGF-β1 increasing gel contraction over a 72 hr period, and 10 ng/mL eIL-10 showing the greatest inhibition of this contraction (Supplementary Figure 2). eTGF-β1 (10 ng/mL) induced contraction of gels laden with all six EDF cell-lines, with a 23% reduction in gel area relative to mock-treated cells (*P* ≤ 0.05, Figure 3B). Treatment with eIL-10 alone (10 ng/mL) did not impact gel area, but did reduce the degree of TGF-β1-induced gel contraction, with only a 16% reduction in gel area relative to the control (*P* ≤ 0.05, Figure 3B). These findings show that the EDFs become contractile in response to eTGF-β1, and that simultaneous exposure to eIL-10 limits the extent of this contraction.

### Equine IL-10 Inhibits eTGF-β1-Induced Production of αSMA and Col 3 in EDFs

**Figure 4:**
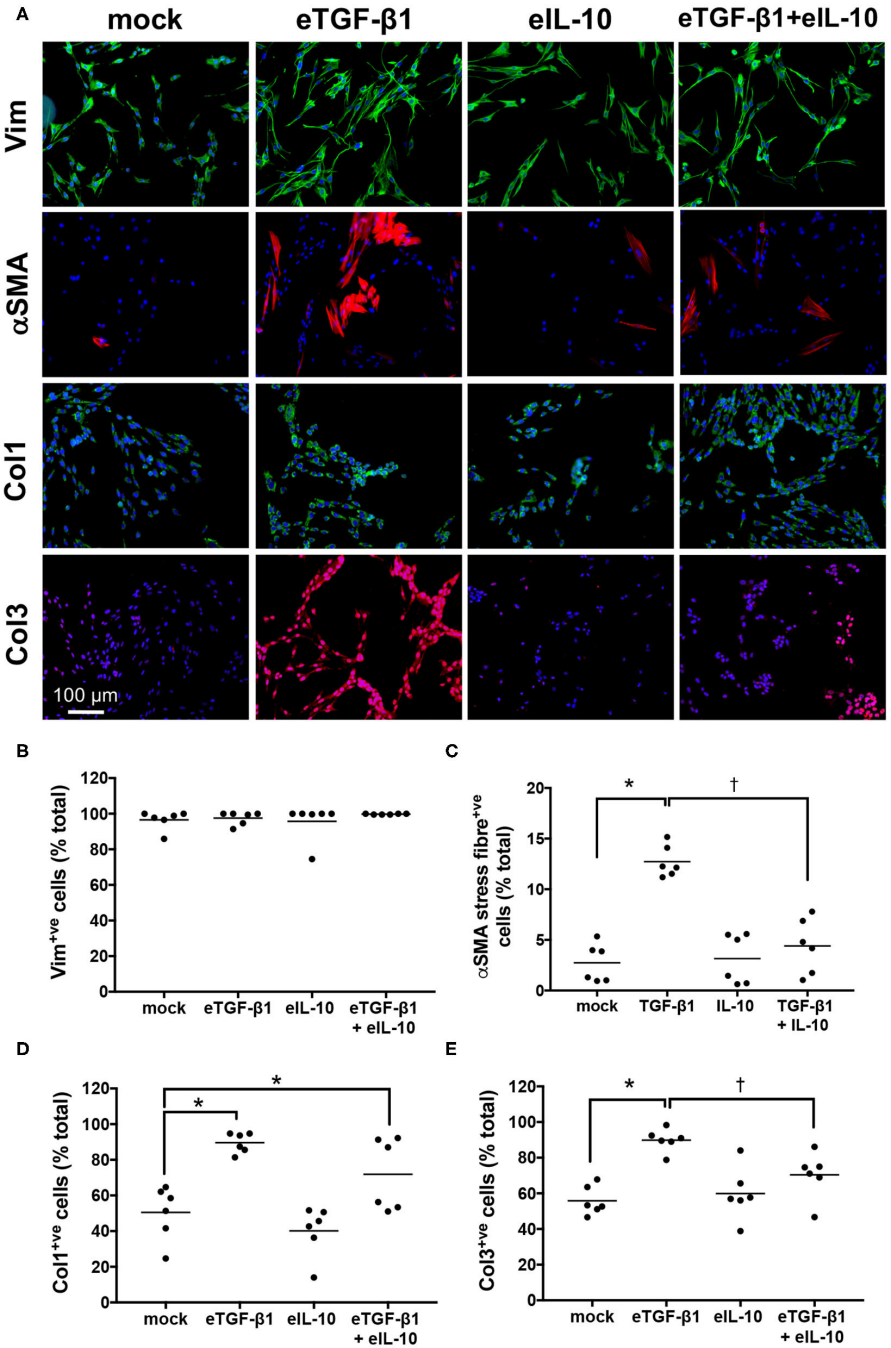
Equine IL-10 inhibits eTGF-β1-induced αSMA and Col 3 production by EDFs. **(A)** Images of representative cell-line H6 cultured for 72 h with eTGF-β1 (10 ng/mL) and/or eIL-10 (10 ng/mL), then stained with DAPI and antibodies against vimentin (Vim), αSMA, Col 1, and Col 3. Scale is as indicated. **(B)** The percentage of DAPI-stained cells co-stained for vimentin. **(C)** The percentage of DAPI-stained cells co-stained for αSMA stress fibers. **(D)** The percentage of DAPI-stained cells co-stained for Col 1 fibers. **(E)** The percentage of DAPI-stained cells co-stained for Col 3 fibers. Each circle represents a cell-line derived from an individual horse, while the line indicates the mean of the six cell-lines. Means significantly different from that of mock or TGF-β1-treated cells are indicated by an asterisk or cross, respectively (*P* ≤ 0.05).

EDF cell-lines were treated with eTGF-β1 and/or eIL-10 and the effects on myofibroblast differentiation and collagen production were assessed using immunofluorescence (Supplementary Figure 3, Figure 4A). The assay was optimized using a single cell-line, with 10 ng/mL eTGF-β1 increasing the percentage of cells showing αSMA^+ve^ stress fibers over a 72 h period, and 10 ng/mL eIL-10 showing the greatest inhibition of this production (Supplementary Figure 3). eTGF-β1 had no effect on the percentage of vimentin^+ve^ cell (Figure 4B), but did increase the percentage of cells positive for αSMA, Col 1 and Col 3 fibers by ~2-fold relative to mock-treated cells (*P* ≤ 0.05, Figures 4C–E). Treatment with eIL-10 alone had no effect on vimentin, αSMA, Col 1, or Col 3 staining, but did prevent the eTGF-β1-induced increase in αSMA and Col 3 fiber production (*P* ≤ 0.05, Figures 4C,E), and dampen the increase in Col 1 staining in three out of the six cell-lines (Figure 4D). These findings indicate that the EDFs differentiate into myofibroblasts and produce type 1 and 3 collagen in response to treatment with eTGF-β1, and that simultaneous treatment with eIL-10 limits these fibrotic processes.

### Equine IL-10 Has Delayed Effects on Fibrotic and Receptor Gene Expression in EDFs

**Figure 5:**
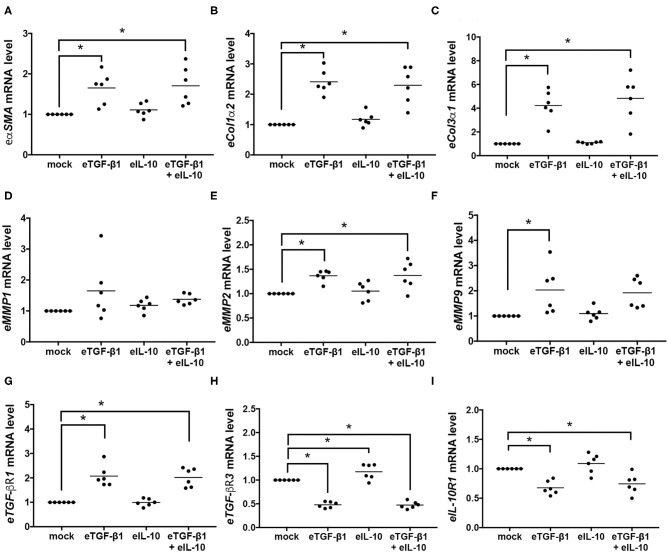
Equine TGF-β1 induces fibrotic and receptor gene expression in EDFs, but eIL-10 has limited effect. Quantitative PCR was used to measure mRNA levels in EDFs after culture with eTGF-β1 (10 ng/mL) and/or eIL-10 (10 ng/mL) for 24 h. Levels of *e*α*SMA* **(A)**, *eCol1*α*2* **(B)**, *eCol3*α*1* **(C)**, *eMMP1* **(D)**, *eMMP2* **(E)**, *eMMP9* **(F)**, *eTGF-*β*R1* **(G)**, *eTGF-*β*R3* **(H)**, and *eIL-10R1* **(I)** are shown relative to *eGAPDH* and mock-treated cells. Each circle represents a cell-line derived from an individual horse, while the line indicates the mean of the six cell-lines. Means significantly different from that of mock-treated cells are indicated by an asterisk (*P* ≤ 0.05).

To evaluate if the changes in EDF contractility, differentiation and collagen production were regulated at a genetic level, quantitative PCR was used to assess mRNA levels in the six cell-lines after 24 h of culture with eTGF-β1 and/or eIL-10 (Figure 5). At that time point, eTGF-β1 increased expression of the fibrotic genes *e*α*SMA, eCol1*α*2, eCol3*α*1, eMMP2*, and *eMMP9* (Figures 5A–C,E,F), but not *eMMP1* (Figure 5D). The addition of eTGF-β1 also resulted in an increase in e*TGF-*β*R1* and a decrease in *eTGF-*β*R3* and *eIL-10R1* expression at 24 h (Figures 5G–I). Treatment with eIL-10 increased the expression of *eTGF-*β*R3* (Figure 5H), and reduced the *eMMP9* expression in response to eTGF-β1 (Figure 5F).

**Figure 6:**
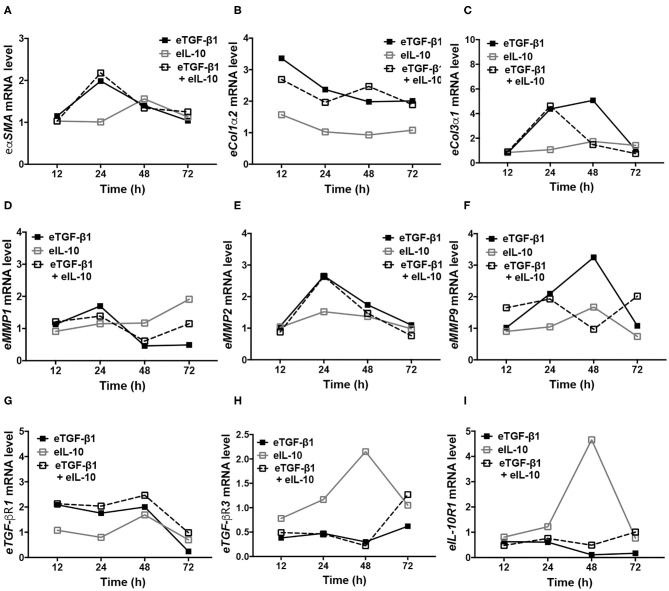
Equine IL-10 has time-dependent effects on fibrotic and receptor regulator expression in eTGF-β1-stimulated and unstimulated EDFs. Quantitative PCR was conducted over 72 h for representative cell-line H6. Levels of *e*α*SMA* **(A)**, *eCol1*α*2* **(B)**, *eCol3*α*1* **(C)**, *eMMP1* **(D)**, *eMMP2* **(E)**, *eMMP9* **(F)**, *eTGF-*β*R1* **(G)**, *eTGF-*β*R3* **(H)**, and *eIL-10R1* **(I)** are shown relative to *eGAPDH* and mock-treated cells.

As gene expression at 24 h did not correspond to the functional and protein changes observed in eTGF-β1 and/or eIL-10 treated EDFs, cumulative changes in gene expression were assessed at various time points in a single cell-line (Figure 6). EDFs derived from horse six was chosen because in all the former analyses, the results for this cell-line were closest to the group mean. The induction of *e*α*SMA* by eTGF-β1 peaked at 24 h, but simultaneous treatment with eIL-10 did not alter its expression at any time point (Figure 6A). eTGF-β1 also increased expression of *eCol1*α*2* at all time points tested (Figure 6B), as well as that of *eCol3*α1 at 24 and 48 h (Figure 6C). eIL-10 treatment reduced the period of time during which *eCol3*α*1* mRNA levels were raised by eTGF-β1 (Figure 6C), but did not impact *eCol1*α*2* expression at any time point (Figure 6B). eTGF-β1 treatment also increased expression of *eMMP1, eMMP2*, and *eMMP9*, with levels peaking after 24, 24, and 48 h, respectively (Figures 6D,E). Although eIL-10 had no effect on *eMMP2* expression (Figure 6E), treatment with this protein reduced eTGF-β1-induced *eMMP9* expression after 48 h (Figure 6F), and increased *eMMP1* expression in the presence and absence of eTGF-β1 after 72 h (Figure 5D). Treatment with eTGF-β1 led to an increase in e*TGF-*β*R1* mRNA from 12 to 48 h, with levels returning to that of mock-treated cells at 72 h, irrespective of whether eIL-10 was present or not (Figure 6G). By contrast, the addition of eTGF-β1 led to a decrease in *eTGF-*β*R3* and *eIL-10R1* expression at all time points (Figures 6H,I). By contrast, eIL-10 treatment increased both *eTGF-*β*R3* and *eIL-10R1* expression at 48 h in the absence of eTGF-β1, and limited the TGF-β1-induced reduction in their expression, particularly after 72 h of treatment (Figures 6H,I). These findings reveal that eTGF-β1 and eIL-10 may have opposing effects on e*TGF-*β*R3* and e*IL-10R1* gene expression in EDFs, and that the effects of eIL-10 treatment on eTGF-β1-induced fibrotic and receptor gene expression may be delayed beyond the original 24 h timepoint tested.

## Discussion

Fibrosis can impact any tissue or organ systems, for example, the skin on the limb of horses where the formation of EGT greatly impedes wound closure. With EGT and other fibroproliferative disorders, chronic inflammation, and expression of TGF-β1 causes activation of myofibroblasts and an imbalance in collagen synthesis and degradation, leading to excessive ECM deposition. IL-10, a potent anti-inflammatory cytokine, has been shown to limit pro-fibrotic responses during tissue repair, directly suppressing myofibroblast differentiation and promoting ECM remodeling. The work to date has utilized IL-10 proteins derived from humans, mice, and viruses, and examined their effects on human, rodent and equine cells or tissues. This study is the first to investigate the effects of recombinant eIL-10 on fibroblasts isolated from the limb skin of horses. Consistent with our hypothesis, eIL-10 exerted anti-fibrotic effects on these cells, reducing eTGF-β1-induced type 3 collagen and αSMA stress fiber production, and their contractility. Preliminary evidence indicates that the anti-fibrotic effects of eIL-10 may be mediated at the transcriptional level, through changes in the expression of ECM components and their degradative enzymes, and selected receptors for eTGF-β1 and eIL-10.

In this study, eIL-10 was shown to suppress the differentiation of fibroblasts into myofibroblasts induced by TGF-β1, as evidenced by a reduction in αSMA stained stress fibers. This did not, however, correspond to a reduction in the transcription of αSMA mRNA, as had previously been reported using human dermal fibroblasts and hypertrophic scar fibroblasts (22). Differences in experimental parameters could account for this discrepancy including cell confluency, presence or absence of serum, and the timing of treatment. eIL-10 may also modulate the turnover of this protein through a mechanism other than gene transcription. For example, IL-10 has been shown to inhibit tumor necrosis factor translation through activation of p38 mitogen-activated protein kinase (MAPK) (26), and the p38 MAPK pathway has been implicated in the degradation of αSMA induced by mechanical stretch and staurosporine (27).

Previous studies have reported that human IL-10 reduces the expression of *Col1*α*2* and *Col3*α*1* in TGF-β1-treated human dermal fibroblasts and in hypertrophic scar fibroblasts (20, 22). In the current study, only the levels of Col 3 were impacted in eTGF-β1-treated EDFs by concurrent treatment with the eIL-10. Again, differences in experimental parameters may account for the selective effects of eIL-10 in EDFs, including the cell density, concentration of ascorbic acid, and culture time frame (28). Alternatively eIL-10 may differentially regulate Col 1 and Col 3 expression, as has been reported with advanced glycation end products that increase Col 1 expression and inhibit Col 3 expression through activation of the p38 MAPK pathway (29). Mechanical stretch has also been shown to downregulate Col 1 mRNA but upregulate Col 3 mRNA in a time-dependent fashion (30).

MMP1 is a collagenase that initiates cleavage of fibrillar collagen, such as Col 1 and Col 3, while gelatinases such as MMP2 and MMP9 regulate the inflammatory response through the cleavage of cytokines (31). Herein, eIL-10 appeared to differentially regulate *eMMP1* and *eMMM9*, increasing and decreasing their mRNA levels, respectively. MMP1 preferentially interacts with Col 3 relative to Col 1 (32), thus increased expression in response to eIL-10 may aid in reducing Col 3 levels in the eTGF-β1-treated EDFs. MMP9, by contrast, activates latent TGF-β1, induces its own expression, and stimulates fibroblast contraction (33), and as such, a reduction following eIL-10 treatment could help suppress eTGF-β1-induced EDF contractility.

To our knowledge, this is the first indication of differential regulation of the TGF-β1 and IL-10 receptors by their ligands. Expression of TGF-βR1 and TGF-βR2 has been detected in equine limb wounds (13), while TGF-βR1, TGF-βR2, and TGF-βR3 (betaregulin) are abundant in human dermal fibroblasts (34). IL-10R1 expression has also been detected in human and equine dermal fibroblasts (12). Here, we observed that eTGF-β1 increased *eTGF-*β*R1*, and decreased *eTGF-*β*R3* and *eIL-10R1* mRNA levels, while eIL-10 treatment of EDFs had the opposite effect. Upon ligand binding, TGF-βR2 forms a complex with, then phosphorylates TGF-βR1, which in turn initiates intracellular signaling (35). Thus, upregulation of *eTGF-*β*R1* is likely to increase TGF-β1-mediated signaling. TGF-βR3, by contrast, has no signaling activity, but has been proposed to modulate healing and scarring process by increasing the affinity of TGFβR2 and inhibins for their receptors (36, 37). The contrasting effects of eTGF-β1 and eIL-10 on *eIL-10R1* mRNA expression observed in this study support the idea that these cytokines may play antagonistic roles during fibrosis. It must be noted however that this observation was generated using a single cell line and findings must be further validated. Future studies should also confirm whether changes in receptor mRNA translate to differences in cell surface protein abundance, and assess whether this affects the actions of other TGF-β family members.

Successful wound healing in humans and horses depends on the contraction of correctly orientated myofibroblasts, and on the cyclic deposition and remodeling of ECM proteins (2). In equine limb wounds, persistent expression of eTGF-β1 likely contributes to myofibroblast activation and spatial disorganization, excess Col 1 production, and EGT formation (9, 14). This is consistent with our findings that EDFs isolated from the limb are responsive to eTGF-β1. As treatment with eIL-10 reduced myofibroblast activation, the deficiency of eIL-10 reported in limb wounds (11) may exacerbate EGT formation, so administration of the recombinant protein could have therapeutic benefit. The clinical relevance of eIL-10 suppression of Col 3 production is however unclear, as the relative abundance of these proteins in equine wounds has not been reported. The chaotic pattern of myofibroblasts within EGT may also relate to a deficiency in platelet-derived growth factor-β which is critical for fibroblast recruitment and increasing ECM stiffness (9, 11, 14, 38). As human IL-10 improved the migratory capacity of TGF-β1-treated myofibroblasts (21), and viral IL-10 increased the migration of fibroblasts derived from the thoracic skin of horses (12), it is plausible that eIL-10 may also modulate EDF trafficking *in vitro*, and in equine limb wounds.

A limitation of this study is that experiments were conducted in a two-dimensional (2D) culture system. Fibroblasts that are grown in this artificially stiff environment are likely to respond to stimuli in a different manner than they would within wounds. The contraction experiments were however performed in a three-dimensional (3D) environment under reduced stiffness. In this scenario, eIL-10 was effective at modulating eTGF-β1-induced effects on EDF contractility. However, both the 2D and 3D assays failed to replicate the complex ECM, regulatory, cellular and hypoxic environment to which the cells are likely exposed *in vivo*. As such, it is difficult to draw conclusions as to whether EDFs would respond in a similar manner to endogenous eTGF-β1 and eIL-10 within a limb wound, or whether TGF-β1 inhibition, or eIL-10 administration, would offer therapeutic benefit in the treatment of equine EGT. This is particularly the case, when the application of recombinant hIL-10 to cutaneous scars produced variable results in randomized control trials (23, 39).

In conclusion, this study aimed to evaluate the effects of recombinant eIL-10 on the pro-fibrotic effects of eTGF-β1 using fibroblasts isolated from the limb skin of horses. Our results indicate that eIL-10 reduces EDF differentiation, contraction and production of ECM. Further investigations are warranted to determine the mechanisms by which the cytokine mediates its anti-fibrotic effects, and to determine whether the recombinant protein can be used to prevent the development of fibroproliferative disorders or promote its resolution in the horse.

## Data Availability Statement

The original contributions generated for the study are included in the article/Supplementary Material, further inquiries can be directed to the corresponding author/s.

## Ethics Statement

Massey University Animal Ethics Committee granted a formal waiver of ethical approval to allow skin samples to be obtained from horses that were euthanized at their owner's request for reasons unrelated to this protocol.

## Author Contributions

LW, CR, and CT designed the study. BA and CR collected the skin samples. GS produced the cell-lines, recombinant protein, and conducted the assays with KS. LW assembled the data, performed the statistical analyses, and prepared the manuscript. LW, CR, CT, and GS edited the manuscript. All authors read and approved the final version of the manuscript.

## Conflict of Interest

The authors declare that the research was conducted in the absence of any commercial or financial relationships that could be construed as a potential conflict of interest.

## Abbreviations

2D: two-dimensional
3D: three-dimensional
ANOVA: analysis of variance
BSA: bovine serum albumin
CD: cluster of differentiation
CLDN: claudin
Col: collagen
DAPI: 4',6-diamidino-2-phenylindole
DMEM: Dulbecco's modified Eagle's medium
e: equine
ECM: extracellular matrix
EDF: equine dermal fibroblasts
EGT: exuberant granulation tissue
ELISA: enzyme-linked immunosorbant assay
FBS: fetal bovine serum
GAPDH: glyceraldehyde-3-phosphate dehydrogenase
h: human
IL: interleukin
MAPK: mitogen-activated protein kinase
MMP: matrix metalloproteinase
NG: neural/glial antigen
PCR: polymerase chain reaction
Pref: preadipocyte factor
R: receptor
RT: room temperature
SEM: standard error of the mean
SMA: smooth muscle actin
TGF: transforming growth factor
Vim: vimentin.
