# Supplementary material for: Anti-fibrotic Actions of Equine Interleukin-10 on Transforming Growth Factor-Beta1-Stimulated Dermal Fibroblasts Isolated From Limbs of Horses
Source: Front Vet Sci. 2020 Sep 18;7:577835. doi: 10.3389/fvets.2020.577835 (PMC7531226; doi:10.3389/fvets.2020.577835)
Supplement: Supplementary file 1 [file Table_1.DOCX]

Supplementary Material

| **Gene name + Accession no.** | **Primer name** | **Primer sequence** | **Product size** | **% efficiency** |
| --- | --- | --- | --- | --- |
| *eGAPDH* | eGAPDH F1 | GCATCGTGGAGGGACTCA | 84 | 105.4 |
| NM_001163856.1 | eGAPDH R1 | GCCACATCTTCCCAGAGG |  |  |
| *eαSMA* | eSMA F2 | TCCGGGGGCACCACCAT | 135 | 93.4 |
| XM_001503035.4 | eSMA R2 | GGAGCCGCCAATCCAGACAG |  |  |
| *eCol3α1* | eCol3a1 F1 | ACGCAAGGCCGTGAGACTA | 63 | 102.2 |
| XM_001917620 | eCol3a1 R1 | TGATCAGGACCACCAACATCA |  |  |
| *eCol1α2* | eCol1a2 F1 | GCACATGCCGTGACTTGAGA | 87 | 99.7 |
| XM_001492939.2 | eCol1a2 R1 | CATCCATAGTGCATCCTTGATTAGG |  |  |
| *eMMP1* | eMMP-1 F3 | CAAATGGACTTCAAGCTGCTTATGAGG | 106 | 99.5 |
| NP_001075316.1 | eMMP-1 R3 | TATCCGTAGAGCACATCCTGCCC |  |  |
| *eMMP2* | eMMP2 F5 | GATAATCTGGACGCCGTGGT | 117 | 94.2 |
| XM_023637007.1 | eMMP2 R5 | GATGCTTCCGAACTTCACGC |  |  |
| *eMMP9* | eMMP9 F2 | CGGCCTACTATGACACCGAC | 71 | 104.7 |
| NM_00111302.1 | eMMP9 R2 | ATTGCCGTCCTGGGTGTAGA |  |  |
| *eTGF-βR1* | eTGFbR1 F10 | TGGCCCTTTTTCAGGAAAGC | 77 | 95.1 |
| XM_023629740.1 | eTGFbR1 R10 | AGCAGACTGGTCCAGCAATG |  |  |
| *eTGF-βR2* | eTGFbR2 F10 | AGAATGACCTGACCTGCTGC | 118 | 99.2 |
| NM_001301147.1 | eTGFbR2 R10 | GGGGCCATGTATCTTGCAGT |  |  |
| *eTGF-βR3* | eTGFbR3 F10 | TGAATGGCTGTGGTACTCGG | 113 | 100.3 |
| XM_02364170.1 | eTGFbR3 F10 | ACCATCTGGCCAACCACTAC |  |  |
| *eIL-10R1* | eIL-10R1 F2 | GGCGGTCTCCTGGGCACAT | 87 | 91.6 |
| XM_001917543.2 | eIL-10R1 R2 | CCTCAGCCCAAGTCACTCAATCG |  |  |
| *eIL-10R2* | eIL-10R2 F3 | CTCGTGGATCATCGCTGTCA | 110 | 94.9 |
| XM_023630053.1 | eIL-10R2 R3 | AGGCGTACTTGGTCTTCACG |  |  |

**Table S1.** Primers used for quantitative PCR analyses.

**Figure S1.** Assessment of non-specific antibody staining. Images of representative cell-line H6 cultured for 72 h then stained with DAPI and isotype or secondary antibody controls. Scale is as indicated.

**Figure S2.** Optimisation of the collagen contraction assay. **(A)** Images of representative Col 1 gels containing cell-line H6, released then cultured with eTGF-β1 (0-10 ng/mL) for up to 72 h. Scale is as indicated. (**B)** Gel area is presented as a percentage of the original size at the time of release. Values are expressed as mean ± SEM of 3 technical replicates. **(C)** Images of representative Col 1 gels containing cell-line H6, released then cultured for 72 h with eTGF-β1 (10 ng/mL) and eIL-10 (0-100 ng/mL). Scale is as indicated. **(D)** Gel area is presented as a percentage of the original size at the time of release. Values are expressed as mean ± SEM of 6 technical replicates.

**Figure S3.** Optimisation of the collagen contraction assay. **(A)** Images of representative cell-line H6 cultured for 72 h with eTGF-β1 (0-10 ng/mL), then stained with DAPI and an antibody against αSMA. Scale is as indicated. **(B)** The percentage of DAPI-stained cells co-stained for αSMA stress fibres. **(C)** representative cell-line H6 cultured for 72 h with eTGF-β1 (10 ng/mL) and eIL-10 (0-100 ng/mL) stained with DAPI and an antibody against αSMA. Scale is as indicated. **(D)** The percentage of DAPI-stained cells co-stained for αSMA stress fibres. Values are expressed as mean ± SEM of 5 technical replicates.
